# High neutrophil counts before endovascular treatment for acute basilar artery occlusion predict worse outcomes

**DOI:** 10.3389/fnagi.2022.978740

**Published:** 2022-09-01

**Authors:** Maofan Liao, Fengli Li, Jinrong Hu, Jie Yang, Deping Wu, Dongjing Xie, Jiaxing Song, Jiacheng Huang, Yan Tian, Weidong Luo, Chengsong Yue, Shuai Liu, Weilin Kong, Li Huang, Wenjie Zi, Fangfei Li

**Affiliations:** Department of Neurology, Xinqiao Hospital and The Second Affiliated Hospital, Army Medical University (Third Military Medical University), Chongqing, China

**Keywords:** leukocyte, neutrophil, NLR, acute basilar artery occlusion, prognosis

## Abstract

**Background and purpose:**

Ischemic stroke is related to inflammation. We investigated leukocyte counts, neutrophil counts, and NLR (neutrophil-to-lymphocyte ratio) to explore their prognostic potential and determine if high neutrophil counts before endovascular treatment (EVT) in patients with acute basilar artery occlusion (BAO) are associated with worse outcomes at 90 days post-EVT.

**Methods:**

Leukocyte and neutrophil counts and NLR were determined in eligible patients from the Acute Basilar Artery Occlusion Study (BASILAR). Patients were divided into four groups according to leukocyte and neutrophil counts and NLR quartiles. The primary outcome was a favorable outcome based on the modified Rankin Scale (mRS: 0–3). The secondary outcome was functional independence (mRS 0–2). The safety outcome was mortality, and an unfavorable outcome was mRS 4–6. Successful reperfusion was mTICI (modified Thrombolysis in Cerebral Infarction) of 2b or 3. All the data were collected within 90 days after EVT.

**Results:**

We enrolled 586 patients in the study. The leukocyte and neutrophil counts and NLR were significantly associated with clinical outcomes in all patients though no effects were seen in some intervals. Of these three parameters, the neutrophil count had the most significant impact, negatively affecting the outcome. The findings were similar in patients who were successfully recanalized.

**Conclusion:**

Higher neutrophil counts predicted worse clinical outcomes 90 days after EVT. This finding supports the deleterious role of inflammation in patients with acute BAO despite EVT or successful recanalization.

## Introduction

Stroke leads to disability and death, causing substantial economic and social burdens (Di Carlo, 2009; Wang et al., 2016). Strokes can be ischemic or hemorrhagic, with the former accounting for 85% of strokes (Rammal and Almekhlafi, 2016). The inflammatory response has been increasingly considered a crucial factor in the events leading to an acute ischemic stroke (AIS) (Iadecola and Anrather, 2011; Kim et al., 2016). Infiltration of leukocytes, especially neutrophils, into the ischemic areas is vital for inflammation (Ishikawa et al., 2004).

After a cerebral infarction, neutrophils are recruited into the ischemic areas representing the early stage of inflammation (Herz et al., 2015). Inflammatory factors such as cytokines, chemokines, and matrix metalloproteinase-9 (MMP-9) released by neutrophils cause oxidative stress, platelet accumulation, and damage to the blood-brain barrier, accelerating the devastation of the brain (Montaner et al., 2001; Castellanos et al., 2003; Shichita et al., 2012; Jickling et al., 2015). Neutrophils are also involved in angiogenesis, neuroplasticity, and neurogenesis, impacting recovery (Perez-de-Puig et al., 2015). Studies using radiolabeled neutrophils have demonstrated their accumulation in the injured zone as the infarct expands. The increasing neutrophil counts are related to the severity and mortality of stroke (Price et al., 2004; Kim et al., 2012; Mo et al., 2013). A dynamic increase in post-stroke neutrophils significantly predicted death or severe disability in patients with AIS treated with recombinant tissue plasminogen activator (r-tPA) for 3 months (Shi et al., 2018). Higher neutrophil counts had a deleterious effect on both the recovery (despite successful recanalization) and recurrence of cerebral infarction in patients with a high-risk TIA or mild ischemic stroke (Zhu et al., 2018b; Boisseau et al., 2019).

In contrast to neutrophils, lymphocyte counts are relatively reduced in AIS patients due to stress-induced corticosteroid response affecting the resolution of inflammation (Jickling et al., 2015). Therefore, the neutrophil to lymphocyte ratio (NLR) might be a promising inflammation biomarker (Zahorec, 2001; Walsh et al., 2005). An elevated NLR is detrimental to the three-month outcome in AIS patients (Brooks et al., 2014; Shi et al., 2018). It is also related to clinical outcomes in stroke patients (Gökhan et al., 2013; Tokgoz et al., 2013; Celikbilek et al., 2014).

However, the published clinical studies have mainly focused on acute anterior circulation ischemic stroke. Hence we sought to evaluate the predictive ability of admission leukocyte count, including neutrophils and NLR, in patients with acute basilar artery occlusion (BAO) treated with endovascular therapy.

## Materials and methods

### Study design and patients selection

Acute Basilar Artery Occlusion Study (BASILAR) was a multicenter prospective clinical registry in China that enrolled patients aged 18 years or older with acute symptomatic and radiological basilar artery occlusion within 24 h of the estimated onset time from January 2014 to May 2019 (Zi et al., 2020). Detailed information on the inclusion and exclusion criteria has been previously published (Zi et al., 2020). In this study, we only included patients who underwent standard medical treatment (SMT) plus endovascular treatment (EVT) (defined as the EVT group). SMT included intravenous thrombolysis, antiplatelet therapy, anticoagulation, or combinations of these treatments. EVT included mechanical thrombectomy, balloon angioplasty, thrombus aspiration, stenting, or a combination of these methods. The therapies each patient underwent relied on the discretion of local interventionists. The research protocol was approved by the ethics committees at all the involved study centers. And informed consent to participate in this study was obtained from the patients.

### Variables and data collection

The data collected included demographic features, stroke risk factors, stroke severity at admission, mechanisms underlying ischemia, occlusion sites, crucial time metrics, and clinical outcomes. The neurological deficit at admission was measured by the National Institutes of Health Stroke Scale (NIHSS) (Brott et al., 1989). The posterior circulation Alberta Stroke Program Early Computed Tomography Score (pc-ASPECTS) indicated the ischemic change at baseline (Puetz et al., 2008). The posterior circulation collateral score (PC-CS) derived from the presence of potential collateral pathways on computed tomography angiography was used to calculate the collateral circulation status (van der Hoeven et al., 2016). The Trial of ORG 10172 in Acute Stroke Treatment (TOAST) classification stratified the stroke etiology (Adams et al., 1993). The modified Thrombolysis in Cerebral Infarction (mTICI) scale was used to grade reperfusion (Zaidat et al., 2013). The included patients (*n* = 586) were divided into four subgroups based on the leukocyte and neutrophil counts and NLR quartiles.

### Outcome measures

We assessed the clinical outcomes at 90 days after EVT using the modified Rankin Scale (mRS) calculated by local neurologists blinded to the research procedures. The primary outcome was mRS 0–3, while the secondary outcome was functional independence with mRS 0–2 at 90 days post-EVT. The safety outcome was the incidence of death within 90 days. At 90 days, mRS 4–6 was considered an unfavorable outcome. Successful reperfusion was measured as mTICI of 2b or 3.

### Statistical analysis

The leukocyte and neutrophil counts and NLR were divided into quartiles as categorical variables. They also were analyzed as continuous variables. The categorical and continuous variables with non-normal distributions were analyzed using the Chi-square and Kruskal–Wallis tests, respectively. The categorical variables were expressed as frequencies and percentages, while the continuous variables were expressed as the median and interquartile range (IQRs). We performed univariable and multivariable logistic regression analyses to assess the associations between essential parameters (as categorical and continuous variables) and clinical outcomes. The following confounding factors were adjusted: age, sex, hypertension, diabetes mellitus, baseline NIHSS score, baseline pc-ASPECTS, PC-CS score, stroke etiology, occlusion sites, and pass. We also examined the relationship between essential parameters and outcomes in a subgroup with successful reperfusion for sensitivity purposes. The same confounders were included.

Generalized linear models were used for subgroup analyses of the neutrophil quartiles to explore the heterogeneity in the associations between clinical characteristics and mRS as a continuous variable at 90 days after EVT in all patients. Models were adjusted for sex, age, baseline NIHSS, baseline pc-ASPECTS, PC-CS score, stroke etiology, occlusion sites, onset to treatment time. The median values were chosen for the threshold age, baseline NIHSS, baseline pc-ASPECTS, PC-CS score, and OTT.

We used Medcalc 20.0.22 to calculate the receiver operating characteristic curve (ROC curve) and establish optimal cutoff points to identify the most effective predictors of clinical outcomes at 90 days post EVT and successful recanalization. The distribution of the mRS scores at 90 days in patients with EVT and successful reperfusion based on the neutrophil quartiles were presented using Microsoft Excel 16.57. STATA 16 (Stata CorpLLC, TX, United States) was used to draw margin plots showing the interactions between neutrophils and clinical outcomes. All statistical analyses were performed using IBM SPSS 25 (IBM Corp., Armonk, NY, United States). The significance level was defined as the two-tailed *p* < 0.05.

## Results

### Baseline characteristics

We included 586 patients who had complete records of leukocyte and neutrophil counts and NLR from the EVT group. Of these patients, 479 (81.7%) were successfully recanalized. In the recanalized patients at 90 days, mRS 0–3 was noted in 178 (37.2%), and functional independence (mRS 0–2) in 151 (31.5%) patients, while 176 (36.7%) patients had died. Supplementary Table 1 summarizes the clinical features of 147, 147, 146, and 146 patients in the four quartiles based on leukocyte and neutrophil counts and NLR. The clinical outcomes were significantly different in the different groups (*p* < 0.05) (Table 1). Supplementary Table 2 presents the basic characteristics of patients with favorable and unfavorable outcomes. Leukocyte and neutrophil counts and NLR as continuous variables significantly affected the outcomes in patients with favorable vs. unfavorable outcomes (*p* < 0.001).

**TABLE 1:** Clinical outcomes of patients undergoing EVT according to quartiles of leukocyte counts (×10^9^/L), neutrophil counts (×10^9^/L), and NLR.

Outcomes, n/total (%)	Leukocyte (×10^9^/L)					Neutrophil (×10^9^/L)					NLR				
	<8.40	8.40–11.06	11.06–13.66	>13.66	*p* value	<6.60	6.60–9.15	9.15–11.96	>11.96	*p* value	<4.96	4.96–7.93	7.93–12.63	>12.63	*p* value
mRS 0-2 at 90d	62(42.2)	42(28.6)	29(19.9)	28(19.2)	<0.001^a^	64(43.5)	39(26.5)	33(22.6)	25(17.1)	<0.001^d^	59(40.4)	42(28.4)	34(23.3)	26(17.8)	<0.001^g^
mRS 0-3 at 90d	71(48.3)	49(33.3)	37(25.3)	32(21.9)	<0.001^b^	74(50.3)	44(29.9)	42(28.8)	29(19.9)	<0.001^e^	66(45.2)	56(37.8)	36(24.7)	31(21.2)	<0.001^h^
Mortality at 90d	50(34.0)	62(42.2)	67(45.9)	81(55.5)	0.003^c^	48(32.7)	66(44.9)	64(43.8)	82(56.2)	0.001^f^	48	64	72	76	0.005^i^

^a^Pairwise comparisons: adjusted p < 0.05 for comparisons between <8.40 and >13.66, between <8.40 and 11.06–13.66.

^b^Pairwise comparisons: adjusted p < 0.05 for comparisons between <8.40 and >13.66, between <8.40 and 11.06–13.66.

^c^Pairwise comparisons: adjusted p < 0.05 for comparisons between <8.40 and >13.66.

^d^Pairwise comparisons.

^e^Pairwise comparisons: adjusted p < 0.05 for comparisons between <6.60 and 6.60–9.15, between <6.60 and 9.15–11.96, between <6.60 and >11.96.

^f^Pairwise comparisons: adjusted p < 0.05 for comparisons between <6.60 and >11.96.

^g^Pairwise comparisons: adjusted p < 0.05 for comparisons between <4.96 and 7.93–12.63, between <4.96 and >12.63.

^h^Pairwise comparisons: adjusted p < 0.05 for comparisons between <4.96 and 7.93–12.63, between <4.96 and >12.63, between 4.96–7.93 and >12.63.

^i^Pairwise comparisons: adjusted p < 0.05 for comparisons between <4.96 and 7.93–12.63, between <4.96 and >12.63. EVT, endovascular treatment; NLR, neutrophil-to-lymphocyte ratio; mRS, modified Rankin Scale.

### Associations of spotlighted parameters with clinical outcomes

#### All patients

In the unadjusted analysis, in patients with mRS 0–2 at 90 days, all the parameters deterred patients from independence (*p* < 0.05) (Table 2). In the adjusted analysis, except for the neutrophil counts in the interval 9.15–11.96 (10^9^/L) (*p* = 0.055), all other parameters had unfavorable effects on patients (Table 2).

**TABLE 2:** Unadjusted and adjusted analyses of neutrophil count (×10^9^/L), leukocyte count (×10^9^/L), NLR with mRS 0–2 at 90days.

Parameters	Cell counts (10^9^/L) in quartiles	Unadjusted analysis		Model 1^†^		Model 2^‡^	
		OR (95% CI)	*p* value	OR (95% CI)	*p* value	OR (95% CI)	*p* value
Leukocyte	<8.40	Reference	<0.001	Reference	0.007	Reference	0.008
	8.40–11.06	0.55(0.34–0.89)	0.015	0.51(0.26–0.97)	0.041	0.52(0.26–1.04)	0.064
	11.06–13.66	0.34(0.20–0.57)	<0.001	0.42(0.21–0.82)	0.011	0.38(0.19–0.77)	0.007
	>13.66	0.33(0.19–0.55)	<0.001	0.32(0.16–0.64)	0.001	0.31(0.15–0.64)	0.002
Neutrophil	<6.60	Reference	<0.001	Reference	0.001	Reference	0.002
	6.60–9.15	0.47(0.29–0.77)	0.002	0.43(0.22–0.83)	0.011	0.50(0.25–0.99)	0.047
	9.15–11.96	0.38(0.23–0.63)	<0.001	0.53(0.27–1.01)	0.055	0.48(0.24–0.96)	0.038
	>11.96	0.27(0.16–0.46)	<0.001	0.23(0.11–0.47)	<0.001	0.22(0.11–0.48)	<0.001
NLR	<4.96	Reference	<0.001	Reference	0.001	Reference	0.001
	4.96–7.93	0.58(0.36–0.95)	0.030	0.46(0.24–0.87)	0.018	0.46(0.23—-0.91)	0.027
	7.93–12.63	0.45(0.27–0.74)	0.002	0.45(0.23–0.89)	0.021	0.41(0.20–0.84)	0.016
	>12.63	0.32(0.19–0.55)	<0.001	0.22(0.11–0.44)	<0.001	0.21(0.10–0.46)	<0.001
Leukocyte, 10^9^/L		0.89(0.84–0.93)	<0.001	0.88(0.83–0.94)	<0.001	0.88(0.82–0.95)	<0.001
Neutrophil, 10^9^/L		0.88(0.83–0.93)	<0.001	0.87(0.81–0.93)	<0.001	0.87(0.81–0.93)	<0.001
NLR		0.95(0.92–0.98)	0.001	0.94(0.91–0.98)	0.001	0.94(0.91–0.98)	0.001

^†^Model 1 adjusted for age, sex, hypertension, diabetes mellitus, baseline NIHSS score, baseline pc-ASPECTS, PC-CS score, stroke etiology, occlusion sites and pass.

^‡^Model 2 excluded patients without successful recanalization and adjusted for confounding factors in model 1.

OR, odds ratio; NLR, neutrophil-to-lymphocyte ratio; mRS, modified Rankin Scale.

In patients with mRS 0–3, the unadjusted analysis found all parameters except NLR at 4.96–7.93 (*p* = 0.200) were negatively associated with favorable outcomes, while in the adjusted analysis, neutrophil counts at 9.15–11.96 (×10^9^/L) and NLR at 4.96–7.93 were not associated with favorable outcomes (Table 3). In the unadjusted analysis, leukocyte counts at 8.40–11.06 (×10^9^/L), neutrophil counts at 9.15–11.96 (×10^9^/L), and NLR at 4.96–7.93 as categorical variables, and NLR as a continuous variable did not affect mortality (Table 4). In the adjusted analysis, except leukocyte count at 8.40–11.06 (×10^9^/L) and 11.06–13.66 (×10^9^/L), neutrophil count at 6.60–9.15 (×10^9^/L) and 9.15–11.96 (×10^9^/L), and NLR at 4.96–7.93, the others promoted death (Table 4).

**TABLE 3:** Unadjusted and adjusted analyses of neutrophil count (x10^9^/L), leukocyte count (×10^9^/L), NLR with mRS 0–3 at 90 days.

Parameters	Cell counts (10^9^/L) in quartiles	Unadjusted analysis		Model 1^†^		Model 2^‡^	
		OR (95% CI)	*p* value	OR (95% CI)	*p* value	OR (95% CI)	*p* value
Leukocyte	<8.40	Reference	<0.001	Reference	0.001	Reference	0.001
	8.40–11.06	0.54(0.33–0.86)	0.009	0.47(0.25–0.87)	0.017	0.44(0.23–0.87)	0.017
	11.06–13.66	0.36(0.22–0.60)	<0.001	0.43(0.23–0.81)	0.009	0.38(0.19–0.75)	0.005
	>13.66	0.30(0.18–0.50)	<0.001	0.28(0.14–0.54)	<0.001	0.26(0.13–0.53)	<0.001
Neutrophil	<6.60	Reference	<0.001	Reference	<0.001	Reference	<0.001
	6.60–9.15	0.42(0.26–0.68)	<0.001	0.34(0.18–0.65)	0.001	0.36(0.18–0.70)	0.003
	9.15–11.96	0.40(0.25–0.65)	<0.001	0.54(0.29–1.01)	0.055	0.48(0.25–0.95)	0.034
	>11.96	0.25(0.15–0.41)	<0.001	0.20(0.10—-0.39)	<0.001	0.18(0.09–0.38)	<0.001
NLR	<4.96	Reference	<0.001	Reference	<0.001	Reference	<0.001
	4.96–7.93	0.74(0.46–1.18)	0.200	0.64(0.34–1.19)	0.159	0.61(0.31–1.19)	0.147
	7.93–12.63	0.40(0.24–0.65)	<0.001	0.35(0.18–0.68)	0.002	0.30(0.15–0.62)	0.001
	>12.63	0.33(0.20–0.55)	<0.001	0.21(0.11–0.42)	<0.001	0.20(0.09–0.41)	<0.001
Leukocyte, 10^9^/L		0.89(0.85–0.93)	<0.001	0.88(0.83–0.94)	<0.001	0.88(0.82–0.94)	<0.001
Neutrophil, 10^9^/L		0.88(0.84–0.93)	<0.001	0.87(0.81–0.92)	<0.001	0.86(0.80–0.92)	<0.001
NLR		0.95(0.92–0.98)	<0.001	0.94(0.90–0.97)	<0.001	0.93(0.90–0.97)	<0.001

^†^Model 1 adjusted for age, sex, hypertension, diabetes mellitus, baseline NIHSS score, baseline pc-ASPECTS, PC-CS score, stroke etiology, occlusion sites and pass.

^‡^Model 2 excluded patients without successful recanalization and adjusted for confounding factors in model 1.

OR, odds ratio; NLR, neutrophil-to-lymphocyte ratio; mRS, modified Rankin Scale.

**TABLE 4:** Unadjusted and adjusted analyses of neutrophil count (×10^9^/L), leukocyte count (×10^9^/L), NLR with mortality at 90 days.

Parameters	Cell counts (10^9^/L) in quartiles	Unadjusted analysis		Model 1^†^		Model 2^‡^	
		OR (95% CI)	*p* value	OR (95% CI)	*p* value	OR (95% CI)	*p* value
Leukocyte	<8.40	Reference	0.003	Reference	0.032	Reference	0.054
	8.40–11.06	1.42(0.88–2.27)	0.150	1.32(0.74–2.34)	0.352	1.31(0.69–2.50)	0.410
	11.06–13.66	1.65(1.03–2.64)	0.039	1.20(0.67–2.16)	0.536	1.29(0.67–2.46)	0.444
	>13.66	2.42(1.51–3.88)	<0.001	2.30(1.28–4.13)	0.005	2.40(1.25–4.62)	0.009
Neutrophil	<6.60	Reference	0.001	Reference	0.006	Reference	0.008
	6.60–9.15	1.68(1.05–2.70)	0.032	1.32(0.74–2.37)	0.352	1.24(0.65–2.39)	0.512
	9.15–11.96	1.61(1.001–2.59)	0.050	1.08(0.60–1.95)	0.786	1.13(0.59–2.18)	0.709
	>11.96	2.64(1.64–4.25)	<0.001	2.55(1.41–4.60)	0.002	2.75(1.42–5.32)	0.003
NLR	<4.96	Reference	0.005	Reference	0.027	Reference	0.017
	4.96–7.93	1.56(0.97–2.50)	0.068	1.66(0.92–3.00)	0.093	1.73(0.88–3.40)	0.115
	7.93–12.63	1.99(1.24–3.19)	0.005	1.82(1.004–3.32)	0.049	1.96(0.99–3.89)	0.053
	>12.63	2.22(1.38–3.56)	0.001	2.50(1.38–4.55)	0.003	2.98(1.51–5.88)	0.002
Leukocyte, 10^9^/L		1.08(1.03–1.13)	<0.001	1.08(1.02–1.14)	0.004	1.09(1.03–1.15)	0.005
Neutrophil, 10^9^/L		1.08(1.04–1.13)	<0.001	1.09(1.03–1.15)	0.002	1.10(1.04–1.17)	0.002
NLR		1.02(1.00–1.04)	0.085	1.02(1.00–1.05)	0.046	1.02(0.996–1.05)	0.098

^†^Model 1 adjusted for age, sex, hypertension, diabetes mellitus, baseline NIHSS score, baseline pc-ASPECTS, PC-CS score, stroke etiology, occlusion sites and pass.

^‡^Model 2 excluded patients without successful recanalization and adjusted for confounding factors in model 1.

OR, odds ratio; NLR, neutrophil-to-lymphocyte ratio; mRS, modified Rankin Scale.

#### Sensitivity analyses in patients with successful recanalization

In all patients who underwent EVT, increasing counts of leukocytes and neutrophils and NLR had detrimental effects on recovery after stroke and promoted mortality. Sensitivity analyses were performed in patients with successful recanalization defined as mTICI of 2b or 3. As seen in all patients, leukocyte and neutrophil counts and NLR as categorical variables in several intervals and continuous variables had an unfavorable influence on some clinical outcomes (Tables 2–4).

**FIGURE 1:**
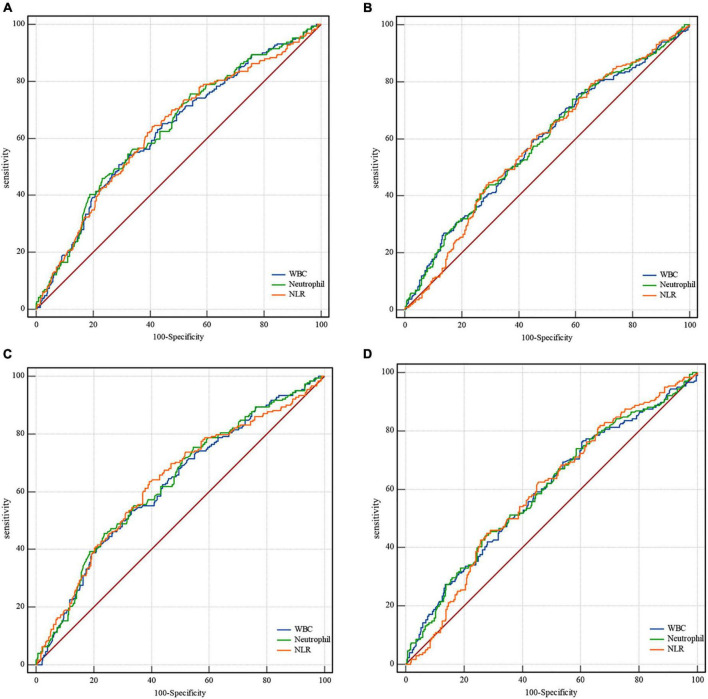
Receiver operating characteristic curves for clinical outcomes. In all patients, the area under the curve for mRS 0–3 is **(A)** 0.627, confidence interval (CI) 0.59–0.67 for leukocytes; 0.634, CI 0.59–0.67 for neutrophils; 0.629, CI 0.59–0.67 for NLR. The area under the curve for mortality in all patients is **(B)** 0.587, CI 0.55–0.63 for leukocytes; 0.589, CI 0.55–0.63 for neutrophils; 0.580, CI 0.54–0.62 for NLR. In successfully recanalized patients, the area under the curve for mRS 0-3 is **(C)** 0.622, CI 0.58-0.67 for leukocytes; 0.630, CI 0.59–0.67 for neutrophils; and 0.630, CI 0.59–0.67 for NLR. The area under the curve for mortality in these patients is **(D)** 0.594, CI 0.55-0.64 for leukocytes; 0.597, CI 0.55–0.64 for neutrophils; and 0.591, CI 0.55–0.64 for NLR. mRS, modified Rankin Scale; CI, confidence interval; NLR, neutrophil-to-lymphocyte ratio.

Based on receiver operating characteristic curves and the area under the curve assessing leukocyte and neutrophil counts and NLR predictive of clinical outcomes, the neutrophil count was found to be the best predictor of mRS 0–2, mRS 0–3, and mortality at 90 days after EVT (Figure 1 and Supplementary Figure 1). The optimal cutoff counts for mRS 0–2, mRS 0–3, and mortality at 90 days were 8.06 × 10^9^/L (55.9% sensitivity and 66.8% specificity), 7.23 × 10^9^/L (46.0% sensitivity and 76.8% specificity), and 7.52 × 10^9^/L (73.9% sensitivity and 41.1% specificity), respectively. Similar results were obtained in successfully recanalized patients with optimal cutoff neutrophil counts of 8.06 × 10^9^/L (55.0% sensitivity and 66.5% specificity), 7.23 × 10^9^/L (45.5% sensitivity and 76.4% specificity), and 10.93 × 10^9^/L (43.8% sensitivity and 72.9% specificity), respectively. Neutrophil counts as dichotomous variables were significantly associated with a lower incidence of mRS 0–2 and mRS 0–3 and a greater risk of mortality in all patients and those who were successfully recanalized (Supplementary Table 3).

Unfavorable outcomes were noted in 301 patients accounting for 62.8% of all the successfully recanalized patients (479). In these patients, high neutrophil counts led to unfavorable outcomes (Supplementary Table 4).

**FIGURE 2:**
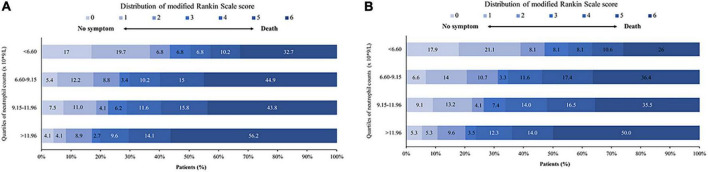
Distribution of mRS at 90 days after EVT. Shown is the distribution of mRS at 90 days for the different neutrophil count quartiles in **(A)** all patients and **(B)** successfully recanalized patients. mRS, modified Rankin Scale; EVT, endovascular treatment.

The rate of favorable outcomes was best with neutrophil count < 6.60 × 10^9^/L. It was lower with higher neutrophil counts, and significantly lower with counts > 11.96 × 10^9^/L (50.3% vs. 29.9% vs. 28.8% vs. 19.9% for subgroups < 6.60 vs. 6.60–9.15 vs. 9.15–11.96 vs >11.96 × 10^9^/L respectively, *p* < 0.001, Figure 2A and Table 1). Similar results were seen in patients with successful recanalization (Figure 2B and Supplementary Table 5).

**FIGURE 3:**
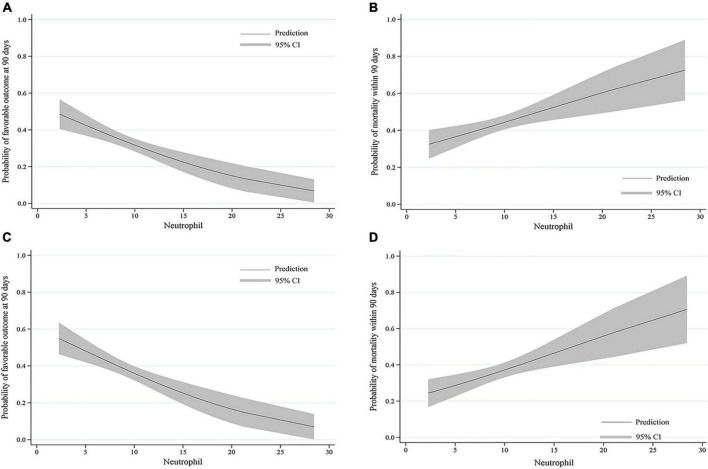
Predicted probability of clinical outcomes by neutrophil count. The curves show a decrease in the estimated probability of favorable outcomes and an increase in the predicted probability of mortality with higher neutrophil counts in **(A,B)** all patients and **(C,D)** patients who were successfully recanalized. The solid line shows the predicted probability of outcomes. The shaded area indicates the 95% confidence interval (CI).

The probability of a favorable outcome declined while mortality increased as the neutrophil count increased in all patients and those who were successfully recanalized (Figure 3).

#### Subgroup analyses

Subgroup analyses in all patients with neutrophil counts >11.96 × 10^9^/L stratified by clinical characteristics were most likely to go from a lower to higher mRS score. However, in male patients, >64 years old, with baseline NIHSS ≤27 or >27, baseline pc-ASPECTS >8, PC-CS score >4 or OTT ≤6 h, neutrophil counts in the intervals 6.60–9.15 (×10^9^/L) or 9.15–11.96 (×10^9^/L) were unfavorable for the decrease of mRS score (Table 5).

**TABLE 5:** Subgroup analyses of mRS score at 90 days in all patients according to quartile of neutrophil counts (×10^9^/L).

Variables	Number of patients	Quartile of neutrophil counts(×10^9^/L)						
		<6.60	6.60–9.15		9.15–11.96		>11.96	
		Adjusted OR (95% CI)	Adjusted OR (95% CI)	*p* value	Adjusted OR (95% CI)	*p* value	Adjusted OR (95% CI)	*p* value
**Sex**								
Male	443	1	0.56(0.33–0.96)	0.034	0.56(0.33–0.96)	0.035	0.27(0.15–0.47)	<0.001
Female	143	1	0.46(0.20–1.09)	0.079	0.83(0.33–2.05)	0.682	0.30(0.10–0.89)	0.030
**Age, years**								
≤64	305	1	0.62(0.32–1.18)	0.146	0.63(0.33–1.21)	0.164	0.32(0.17–0.62)	0.001
>64	281	1	0.51(0.27–0.95)	0.035	0.64(0.33–1.23)	0.181	0.24(0.11–0.51)	<0.001
**Baseline NIHSS**								
≤27	304	1	0.62(0.35–1.07)	0.087	0.50(0.27–0.90)	0.021	0.27(0.15–0.49)	<0.001
>27	282	1	0.40(0.19–0.84)	0.015	0.88(0.43–1.79)	0.722	0.30(0.14–0.65)	0.002
**Baseline pc–ASPECTS**								
≤8	378	1	0.67(0.38–1.19)	0.174	0.77(0.44–1.36)	0.369	0.27(0.14–0.51)	<0.001
>8	204	1	0.36(0.18–0.74)	0.005	0.40(0.18–0.85)	0.017	0.32(0.16–0.65)	0.002
**PC-CS score**								
≤4	293	1	0.70(0.36–1.34)	0.281	0.87(0.43–1.77)	0.708	0.34(0.17–0.69)	0.003
>4	292	1	0.50(0.27–0.93)	0.029	0.63(0.34–1.14)	0.125	0.31(0.16–0.59)	<0.001
**Stroke etiology**								
LAA	382	1	0.62(0.35–1.09)	0.099	0.75(0.43–1.31)	0.311	0.31(0.17–0.55)	<0.001
CE	152	1	0.51(0.22–1.18)	0.116	0.70(0.28–1.77)	0.455	0.25(0.10–0.66)	0.005
Other	52	1	1.25(0.19–8.16)	0.815	1.97(0.32–12.29)	0.467	1.38(0.22–8.84)	0.735
**Occlusion sites**								
Distal BA	194	1	0.49(0.24–1.00)	0.050	0.75(0.34–1.65)	0.473	0.16(0.06–0.41)	<0.001
Middle BA	185	1	0.49(0.22–1.12)	0.090	0.74(0.31–1.74)	0.485	0.30(0.12–0.73)	0.008
Proximal BA	98	1	0.66(0.20–2.19)	0.495	0.38(0.12–1.26)	0.115	0.26(0.08–0.83)	0.023
VA-V4	109	1	0.84(0.23–3.05)	0.788	0.54(0.18–1.65)	0.281	0.43(0.14–1.32)	0.139
**OTT, h**								
≤6	420	1	0.55(0.33–0.92)	0.023	0.62(0.37–1.05)	0.076	0.32(0.18–0.54)	<0.001
>6	166	1	0.72(0.29–1.77)	0.473	1.25(0.49–3.18)	0.639	0.29(0.11–0.78)	0.014

Models adjusted for sex, age, baseline NIHSS score, baseline pc-ASPECTS, PC-CS score, stroke etiology, occlusion sites and OTT.

OR, odds ratio; mRS, modified Rankin Scale; NIHSS, National Institutes of Health Stroke Scale; pc-ASPECTS, posterior circulation Alberta Stroke Program Early Computed Tomography Score; PC-CS, posterior circulation collateral score; LAA, large artery atherosclerosis; CE, cardioembolic; BA, basilar artery; VA-V4, vertebral artery-V4 segment; OTT, onset to treatment.

## Discussion

We evaluated the effects of leukocytes, neutrophils, and the ratio (NLR) on clinical outcomes in patients with acute BAO who underwent EVT. Although all three parameters affected outcomes, neutrophils most significantly impacted mRS 0–2, mRS 0–3, and mortality within 90 days after EVT. The optimal cutoff neutrophil counts that best discriminated these clinical outcomes were 8.06 × 10^9^/L (mRS 0–2), 7.23 × 10^9^/L (mRS 0–3), and 7.52 × 10^9^/L (mortality). Higher neutrophil counts were associated with more unfavorable outcomes.

This nationwide multicenter study in China included patients who underwent EVT for BAO. Blood samples were drawn before treatment. Since neutrophil and leukocyte counts are estimated as a part of the routine blood work, evaluating them as predictors of inflammation and clinical outcomes did not incur additional costs.

However, some significant limitations of the study should be taken into consideration. First, neutrophil counts were determined only at admission. The kinetics of neutrophils during the stroke and their influence on the clinical outcomes should be assessed. Previous studies have shown that an increase in leukocytes or neutrophils was significantly associated with unfavorable outcomes in patients with ischemic stroke (Grau et al., 2004; Shi et al., 2018). However, one study indicated that neutrophils taken within 12 h after the onset of stroke affected the ischemic infarction size measured by diffusion-weighted MR imaging (DWI), suggesting an association between early proinflammation and deterioration of AIS (Buck et al., 2008). Second, only mRS was evaluated as a clinical outcome. Whether neutrophils affect other clinical outcomes needs to be evaluated. Third, the time of venipuncture could influence the serum sample. In our study, the time from stroke onset to blood sample collection was not documented. Fourth, there might be residual bias because of a pre-existing infection, trauma, or tumor. A pre-existing infection has been shown to result in worse outcomes in young adults with AIS (Heikinheimo et al., 2013). Finally, it was hard for us to validate a cause-result relationship between neutrophil counts and clinical outcomes because of the observational nature of the study.

Our study indicated that higher neutrophils predicted worse outcomes regardless of the underlying cause for the increase in neutrophils. Inflammatory factors released by neutrophils play an essential role in increasing the neutrophil counts, leading to endothelium damage and platelet aggregation impairing the microcirculation (Ernst et al., 1987; Borissoff and ten Cate, 2011; Döring et al., 2015). In addition, neutrophil recruitment and parietal fibrin deposition are detected in the microvasculature during AIS, termed downstream microvascular thromboinflammation (DMT), which contributes to incomplete reperfusion (del Zoppo and Mabuchi, 2003; Desilles et al., 2015, 2017, 2018). Cerebral thrombus consists of neutrophils and NETs (neutrophil extracellular traps), and the NETs affect tPA-induced reperfusion resistance (Laridan et al., 2017; Ducroux et al., 2018). Animal studies evidenced that pharmacologically induced neutropenia has a neuroprotective role in rats with experimental ischemic stroke (Pétrault et al., 2005). Similar findings were reported in experimental models investigating neutrophilic inhibition through the neutrophil inhibitory factor (Jiang et al., 1995). Consistent with these reports, we found that neutrophils negatively affected clinical outcomes despite successful recanalization. Moreover, the disruption of the blood–brain barrier (BBB) and transmigration of neutrophils reinforced each other (Tang et al., 2014; Jickling et al., 2015).

Our findings are consistent with previous reports as follows suggesting a significant relationship between neutrophils and clinical outcomes in patients with anterior and posterior circulation or only anterior circulation ischemic stroke. Leukocytes, neutrophils, and NLR were positively associated with clinical outcomes in patients treated with intravenous thrombolysis (Malhotra et al., 2018). Similarly elevated neutrophil counts promoted worse outcomes and the recurrence of stroke (Maestrini et al., 2015; Zhu et al., 2018a). However, these studies evaluated neutrophils as a continuous variable or one of the binary categorical variables. In contrast, we divided the neutrophil counts into quartiles and found that neutrophils played the most crucial role in predicting clinical outcomes. However, neutrophil counts in the interval 9.15–11.96 (×10^9^/L) showed no association with mRS 0–2 and mRS 0–3. This could be due to uncertain confounders leading to a lack of statistical power, affecting the actual association between this interval and outcomes. Moreover, we did not consider the seasonal and diurnal fluctuations in neutrophil counts and pre-existing infections, which need to be further studied (Fisch and Freedman, 1975; Woodhouse et al., 1994). Published studies focusing on the leukocyte and neutrophil quartiles and other subgroups of leukocytes have reported similar effects of neutrophils on the recurrence of ischemic events (Grau et al., 2004; Zhu et al., 2018b).

Based on our findings, neutrophils could be a valuable 90-days prognostic factor in patients with acute BAO treated with EVT. However, we are not suggesting neutrophil counts at admission as the most vital determinant of whether a patient should receive EVT or not. The prognostic potential of neutrophils still needs to be further validated in various clinical settings.

In conclusion, leukocytes, neutrophils, and NLR were significantly associated with a lower likelihood of favorable outcomes and increased odds of unfavorable outcomes. Among them, neutrophils contributed most to these clinical outcomes. Additionally, neutrophils might serve as potential neuroprotective targets in these patients.

## Data availability statement

The original contributions presented in this study are included in the article/Supplementary material, further inquiries can be directed to the corresponding authors.

## Ethics statement

The studies involving human participants were reviewed and approved by the Ethics Committee of the Xinqiao Hospital, Army Medical University, in Chongqing, China, and each subcenter. The patients/participants provided their written informed consent to participate in this study.

## Author contributions

ML, FLL, and JRH: drafting/revision of the manuscript for content, including medical writing for content, major role in the acquisition of data, and analysis or interpretation of data. JY, DW, DX, JCH, YT, WL, CY, SL, WK, and LH: major role in the acquisition of data and analysis or interpretation of data. WZ and FFL: drafting/revision of the manuscript for content, including medical writing for content and analysis or interpretation of data. All authors contributed to the article and approved the submitted version.
