# Supplementary material for: High neutrophil counts before endovascular treatment for acute basilar artery occlusion predict worse outcomes
Source: Front Aging Neurosci. 2022 Sep 1;14:978740. doi: 10.3389/fnagi.2022.978740 (PMC9475290; doi:10.3389/fnagi.2022.978740)
Supplement: Supplementary file 1 [file Data_Sheet_1.PDF]

Supplementary Appendix

This appendix has been provided by the authors to give readers additional information about their work

eTable 1. Baseline Characteristics of patients undergoing EVT according to quartiles of leukocyte counts ($x10^9$/L), neutrophil counts ($x10^9$/L) and NLR

eTable 2. Baseline Characteristics of groups between favorable outcomes and unfavorable outcomes

eTable 3. Unadjusted analyses and adjusted analyses of neutrophil count ($x10^9$/L) as dichotomous variables with clinical outcomes

eTable 4. Unadjusted and adjusted analyses of neutrophil count ($x10^9$/L), leukocyte count ($x10^9$/L), NLR with unfavorable outcomes in successfully recanalized patients

eTable 5. Frequencies of quartile of neutrophil counts for favorable outcomes in patients with successful recanalization

eFigure 1 Receiver operating characteristic curves for clinical outcomes

eTable 1 Baseline Characteristics of patients undergoing EVT according to quartiles of leukocyte counts (x10^9/L), neutrophil counts (x10^9/L) and NLR

	Leukocyte (x10^9/L)					Neutrophil (x10^9/L)					NLR				
Characteristics	<8.40	8.40-11.06	11.06-13.66	>13.66	P value	<6.60	6.60-9.15	9.15-11.96	>11.96	P value	<4.96	4.96-7.93	7.93-12.63	>12.63	P value
Age, median (IQR), y	66(59-74)	65(59-74)	63(57-72)	61(52-71)	0.001	65(59-74)	66(58-74)	63(57-72)	61(52-71)	0.003	63(57-71)	66(57-74)	65(55-73)	64(55-71)	0.634
Male, n/total (%)	102(69.4)	109(74.1)	110(75.3)	122(83.6)	0.041	103(70.1)	105(71.4)	114(78.1)	121(82.9)	0.037	100(68.5)	114(77.0)	118(80.8)	111(76.0)	0.096
Medical history, n/total (%)															
Hypertension	100(68.0)	96(65.3)	103(70.5)	104(71.2)	0.685	100(68.0)	99(67.3)	101(69.2)	103(70.5)	0.940	102(69.9)	105(70.9)	95(65.1)	101(69.2)	0.717
Hyperlipidemia	44(29.9)	35(23.8)	53(36.3)	63(43.2)	0.003	48(32.7)	37(25.2)	51(34.9)	59(40.4)	0.048	55(37.7)	45(30.4)	50(34.2)	45(30.8)	0.517
Diabetes mellitus	36(24.5)	31(21.1)	33(22.6)	34(23.3)	0.918	36(24.5)	31(21.1)	36(24.7)	31(21.2)	0.809	44(30.1)	29(19.6)	31(21.1)	30(20.5)	0.115
Smoking	48(32.7)	57(38.8)	51(34.9)	57(39.0)	0.609	53(36.1)	53(36.1)	53(36.3)	54(37.0)	0.998	58(39.7)	52(35.1)	63(43.2)	40(27.4)	0.032
Ischemic stroke	28(19.0)	35(23.8)	35(24.0)	32(21.9)	0.720	31(21.1)	36(24.5)	29(19.9)	34(23.3)	0.774	32(21.9)	36(24.3)	30(20.5)	32(21.9)	0.889
Atrial fibrillation	40(27.2)	34(23.1)	25(17.1)	22(15.1)	0.040	34(23.1)	40(27.2)	25(17.1)	22(15.1)	0.040	29(19.9)	32(21.6)	33(22.6)	27(18.5)	0.827
Baseline NIHSS score, median (IQR)	22(13-31)	28(16-34)	28(21-33)	29(18-34)	0.001	22(13-31)	28(16-34)	28(18-33)	29(19-34)	0.001	23(14-31)	28(16-34)	28(19-34)	28(18-33)	0.004
Baseline pc-ASPECTS, median (IQR)	8(7-10)	8(7-9)	8(6-9)	8(7-9)	0.008	8(7-10)	8(7-9)	8(6-9)	8(7-9)	0.010	8(7-9)	8(7-9)	8(7-9)	8(7-9)	0.835
PC-CS score, median (IQR)	5(3-6)	4(4-6)	5(3-6)	4(2-6)	0.222	5(3-6)	4(3-6)	5(3-6)	4(2-6)	0.131	5(4-6)	5(3-6)	4(3-6)	4(2-6)	0.005

Body temperature, median (IQR), ℃	36.5(36.5-36.8)	36.6(36.5-36.8)	36.5(36.5-36.8)	36.7(36.5-36.9)	0.032	36.5(36.5-36.8)	36.6(36.5-36.8)	36.7(36.5-36.9)	36.6(36.5-36.9)	0.029	36.5(36.5-36.8)	36.6(36.5-36.8)	36.6(36.5-36.81)	36.7(36.5-37.0)	0.027
SBP, median (IQR), mm Hg	146(131-162)	154(133-170)	146(129-167)	150(135-164)	0.252	149(134-164)	150(130-166)	147(130-168)	150(135-165)	0.801	147(134-163)	150(130-170)	150(129-164)	150(137-166)	0.627
DBP, median (IQR), mm Hg	83(75-94)	87(78-98)	84(75-96)	85(78-100)	0.104	82(75-94)	86(78-98)	84(75-95)	86(78-100)	0.061	83(77-94)	84(76-98)	84(75-95)	89(80-100)	0.127
Stroke etiology, n/total (%)															
LAA	88(59.9)	91(61.9)	98(67.1)	105(71.9)	0.029	92(62.6)	90(61.2)	97(66.4)	103(70.5)	0.100	98(67.1)	95(64.2)	97(66.4)	92(63.0)	0.461
CE	50(34.0)	44(29.9)	30(20.5)	28(19.2)		47(32.0)	44(29.9)	34(23.3)	27(18.5)		41(28.1)	40(27.0)	35(24.0)	36(24.7)	
Other	9(6.1)	12(8.2)	18(12.3)	13(8.9)		8(5.4)	13(8.8)	15(10.3)	16(11.0)		7(4.8)	13(8.8)	14(9.6)	18(12.3)	
Occlusion sites, n/total (%)															
Distal BA	55(37.4)	64(43.5)	42(28.8)	33(22.6)	0.002	58(39.5)	62(42.2)	39(26.7)	35(24.0)	0.002	53(36.3)	50(33.8)	46(31.5)	45(30.8)	0.201
Middle BA	44(29.9)	47(32.0)	47(32.2)	47(32.2)		42(28.6)	49(33.3)	49(33.6)	45(30.8)		52(35.6)	49(33.1)	49(33.6)	35(24.0)	
Proximal BA	26(17.7)	19(12.9)	28(19.2)	25(17.1)		26(17.7)	21(14.3)	24(16.4)	27(18.5)		21(14.4)	25(16.9)	24(16.4)	28(19.2)	
VA-V4	22(15.0)	17(11.6)	29(19.9)	41(28.1)		21(14.3)	15(10.2)	34(23.3)	39(26.7)		20(13.7)	24(16.2)	27(18.5)	38(26.0)	
Treatment profiles															
ITV, n/total (%)	24(16.3)	32(21.8)	27(18.5)	26(17.8)	0.673	25(17.0)	31(21.1)	28(19.2)	25(17.1)	0.779	27(18.5)	32(21.6)	23(15.8)	27(18.5)	0.642
	182(69-338)	200(108-350)	215(85-344)	212(90-401)	0.388	164(60-313)	191(108-347)	225(85-358)	220(105-401)	0.075	151(54-281)	225(89-346)	214(99-395)	241(110-406)	0.002

OTI, median (IQR), min															
	225(109-385)	245(141-387)	251(138-376)	258(134-432)	0.366	195(107-339)	245(151-385)	261(136-394)	260(155-432)	0.029	195(105-319)	263(134-379)	249(136-428)	286(159-452)	0.002
OTT, median (IQR), min															
	407(310-591)	420(315-592)	485(362-703)	459(341-659)	0.014	384(304-549)	442(345-600)	487(335-704)	470(348-674)	0.001	401(311-517)	450(319-620)	418(311-632)	501(377-767)	<0.001
Onset to recanalization time, median (IQR), min															
	100(60-139)	91(66-138)	118(80-151)	107(77-168)	0.002	99(66-136)	98(66-138)	115(77-152)	111(73-169)	0.007	104(71-141)	100(69-138)	106(66-153)	113(86-167)	0.054
Puncture to recanalization time, median (IQR), min															
Pass, n/total (%)															
	131(94.9)	125(94.0)	125(96.2)	127(94.8)	0.883	130(95.6)	127(95.5)	122(92.4)	129(96.3)	0.486	119(93.7)	135(97.1)	131(96.3)	123(92.5)	0.260
Pass0-3															

Abbreviations: EVT, endovascular treatment; NLR, neutrophil-to-lymphocyte ratio; NIHSS, National Institutes of Health Stroke Scale; pc-ASPECTS, posterior circulation Alberta Stroke Program Early Computed Tomography Score; PC-CS, posterior circulation collateral score; SBP, systolic blood pressure; DBP, diastolic blood pressure; LAA, large artery atherosclerosis; CE, cardioembolic; BA, basilar artery; VA-V4, vertebral artery-V4 segment; IVT, intravenous thrombolysis; OTI, Onset to imaging diagnosis time; OTT, onset to treatment.

eTable 2 Baseline Characteristics of groups between favorable outcomes and unfavorable outcomes

Characteristics	All(n=586)	Favorable outcomes(n=189)	Unfavorable outcomes(n=397)	P value
Age, median (IQR), y	64(56-73)	63(55-71)	65(57-73)	0.067
Male, n/total (%)	443(75.6)	138(73.0)	305(76.8)	0.315
Medical history, n/total (%)				
Hypertension	403(68.8)	128(67.7)	275(69.3)	0.706
Hyperlipidemia	195(33.3)	68(36.0)	127(32)	0.338
Diabetes mellitus	134(22.9)	29(15.3)	105(26.4)	0.003
Smoking	213(36.3)	72(38.1)	141(35.5)	0.544
Ischemic stroke	130(22.2)	35(18.5)	95(23.9)	0.141
Atrial fibrillation	121(20.6)	45(23.8)	76(19.1)	0.192
Baseline NIHSS score, median (IQR)	27(17-33)	18(10.5-27)	30(21-34)	<0.001
Baseline pc-ASPECTS, median (IQR)	8(7-9)	9(8-10)	7(6-9)	<0.001
PC-CS score, median (IQR)	4(3-6)	5(4-6)	4(3-6)	<0.001
Body temperature, median (IQR), ℃	36.6(36.5-36.8)	36.5(36.5-36.8)	36.6(36.5-36.8)	0.040
Blood pressure on admission, median (IQR), mm Hg				
Systolic	149(133-166)	146(130-161)	150(134-168)	0.050
Diastolic	84(76-97)	84(75.5-94.5)	85(76.8-98)	0.277
Stroke etiology, n/total (%)				
LAA	382(65.2)	109(57.7)	273(68.8)	
CE	152(25.9)	60(31.7)	92(23.2)	0.031
Other	52(8.9)	20(10.6)	32(8.1)	
Occlusion sites, n/total (%)				
Distal BA	194(33.1)	83(43.9)	111(28.0)	
Middle BA	185(31.6)	50(26.5)	135(34.0)	0.002

Proximal BA	98(16.7)	29(15.3)	69(17.4)	
VA-V4	109(18.6)	27(14.3)	82(20.7)	
Treatment profiles				
Intravenous thrombolysis, n/total (%)	109(18.6)	36(19.0)	73(18.4)	0.848
Onset to imaging diagnosis time, median (IQR), min	208.5(85-355.3)	200(85.5-316.5)	210(85-363.5)	0.710
Onset to treatment, median (IQR), min	245.5(129.8-390)	244(132-354.5)	246(128.5-407.7)	0.800
Pass, n/total (%)				
Pass0-3	508(95.0)	173(98.3)	335(93.3)	0.013
Pass4-6	27(5.0)	3(1.7)	24(6.7)	
Leukocyte, median (IQR), 10^9/L	11.06(8.40-13.66)	9.60(7.63-12.61)	11.60(9.10-14.44)	<0.001
Neutrophil, median (IQR), 10^9/L	9.15(6.60-11.96)	7.80(5.96-10.22)	9.84(7.3-12.50)	<0.001
NLR, median (IQR)	7.93(4.96-12.63)	6.26(3.95-10.03)	9.06(5.68-13.51)	<0.001

Abbreviations: NIHSS, National Institutes of Health Stroke Scale; pc-ASPECTS, posterior circulation Alberta Stroke Program Early Computed Tomography Score; PC-CS, posterior circulation collateral score; LAA, large artery atherosclerosis; CE, cardioembolic; BA, basilar artery; VA-V4, vertebral artery-V4 segment.

eTable 3 Unadjusted analyses and adjusted analyses of neutrophil count (x10^9/L) as dichotomous variables with clinical outcomes

		Unadjusted analyses		Adjusted analyses	
Groups	Neutrophil cell counts(10^9/L)	OR (95% CI)	P value	OR (95% CI)	P value
All patients	>8.06 vs ≤8.06	0.37(0.25-0.54)	<0.001	0.43(0.25-0.74)	0.002
	>7.23 vs ≤7.23	0.35(0.25-0.51)	<0.001	0.36(0.21-0.62)	<0.001
	>7.52 vs ≤7.52	1.97(1.38-2.81)	<0.001	1.64(1.03-2.62)	0.036
Successful recanalization	>8.06 vs ≤8.06	0.41(0.28-0.61)	<0.001	0.44(0.25-0.76)	0.004
	>7.23 vs ≤7.23	0.37(0.25-0.55)	<0.001	0.35(0.20-0.61)	<0.001
	>10.93 vs ≤10.93	2.10(1.42-3.10)	<0.001	2.02(1.22-3.33)	0.006

Models adjusted for age, sex, hypertension, diabetes mellitus, baseline NIHSS score, baseline pc-ASPECTS, PC-CS score, stroke etiology, occlusion sites and pass.

Abbreviations: OR, odds ratio.

eTable 4 Unadjusted and adjusted analyses of neutrophil count (x10^9/L), leukocyte count (x10^9/L), NLR with unfavorable outcomes in successfully recanalized patients

	Cell counts(10^9/L) in quartiles	Unadjusted analysis		Adjusted analyses	
Parameters		OR (95% CI)	P value	OR (95% CI)	P value
Leukocyte	<8.40	Reference	<0.001	Reference	0.001
	8.40-11.06	1.99(1.20-3.30)	0.008	2.25(1.16-4.39)	0.017
	11.06-13.66	2.64(1.56-4.47)	<0.001	2.63(1.34-5.17)	0.005
	>13.66	3.20(1.85-5.54)	<0.001	3.89(1.91-7.92)	<0.001
Neutrophil	<6.60	Reference	<0.001	Reference	<0.001
	6.60-9.15	2.33(1.39-3.90)	0.001	2.80(1.42-5.51)	0.003
	9.15-11.96	2.41(1.44-4.05)	0.001	2.06(1.05-4.04)	0.034
	>11.96	3.98(2.28-6.97)	<0.001	5.46(2.63-11.31)	<0.001
NLR	<4.96	Reference	<0.001	Reference	<0.001
	4.96-7.93	1.46(0.88-2.42)	0.149	1.65(0.84-3.22)	0.147
	7.93-12.63	2.76(1.61-4.74)	<0.001	3.29(1.61-6.74)	0.001
	>12.63	3.20(1.84-5.56)	<0.001	5.07(2.42-10.63)	<0.001
Leukocyte, 10^9/L		1.12(1.06-1.18)	<0.001	1.14(1.07-1.22)	<0.001
Neutrophil, 10^9/L		1.13(1.07-1.19)	<0.001	1.16(1.09-1.24)	<0.001
NLR		1.05(1.02-1.08)	0.002	1.07(1.03-1.11)	<0.001

Models adjusted for age, sex, hypertension, diabetes mellitus, baseline NIHSS score, baseline pc-ASPECTS, PC-CS score, stroke etiology, occlusion sites and pass.

Abbreviations: OR, odds ratio; NLR, neutrophil-to-lymphocyte ratio.

eTable 5 Frequencies of quartile of neutrophil counts for favorable outcomes in patients with successful recanalization

Parameter	Cell counts(10^9/L) in quartile	Frequencies	P value
Neutrophil	<6.60	68(55.3)	<0.001
	6.60-9.15	42(34.7)	
	9.15-11.96	41(33.9)	
	>11.96	27(23.7)	

eFigure 1 Receiver operating characteristic curves for clinical outcomes

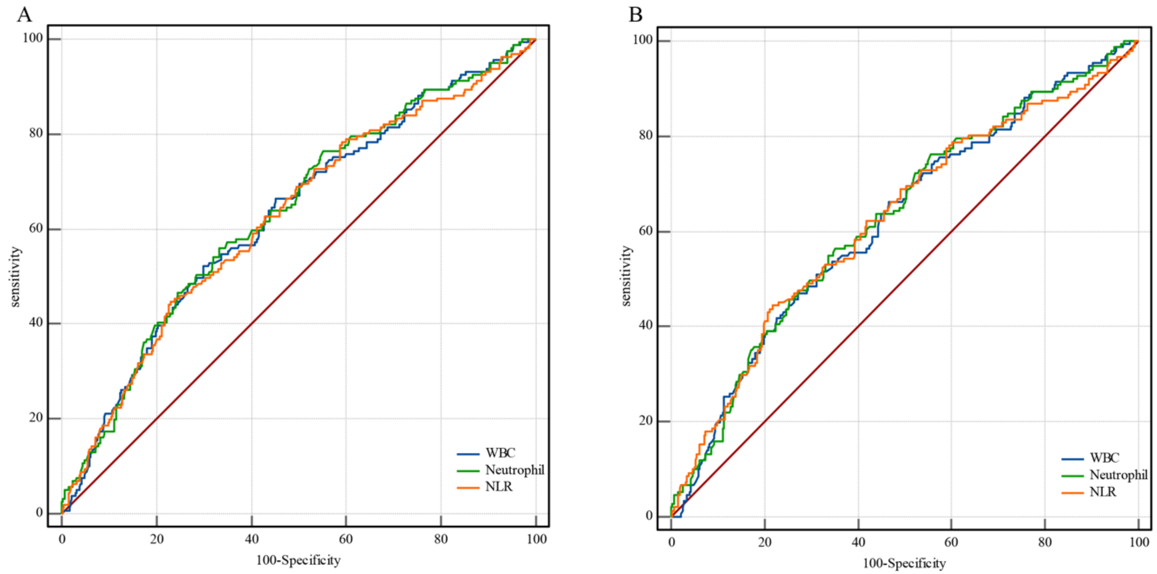

In all patients, the area under the curve for mRS 0-2 is (A) 0.629, confidence interval (CI) 0.59-0.67 for leukocytes; 0.634, CI 0.59-0.67 for neutrophils; and 0.624, CI 0.58-0.66 for NLR.

In successfully recanalized patients, the area under the curve for mRS 0-2 is (B) 0.622, CI 0.58-0.67 for leukocytes; 0.628, CI 0.58-0.67 for neutrophils; and 0.624, CI 0.58-0.67 for NLR.

Abbreviations: mRS, modified Rankin Scale; CI, confidence interval; NLR, neutrophil-to-lymphocyte ratio.
